# Immune checkpoint inhibitor induced colitis and arthritis: A case report

**DOI:** 10.1097/MD.0000000000036334

**Published:** 2023-12-08

**Authors:** Rong-Xin Xie, Yu-Bao Xue, Xin-Yu Ci, Mei-Juan Zhang

**Affiliations:** a Department of Gastroenterology, The First Affiliated Hospital of Shandong First Medical University & Shandong Provincial Qianfoshan Hospital, Shandong First Medical University, Jinan, Shandong, China; b Department of Gastroenterology, The First Affiliated Hospital of Shandong First Medical University & Shandong Provincial Qianfoshan Hospital, Jinan, Shandong, China; c Department of Health Management, The First Affiliated Hospital of Shandong First Medical University & Shandong Provincial Qianfoshan Hospital, Shandong Engineering Laboratory for Health Management, Jinan, Shandong, China.

**Keywords:** arthritis, camrelizumab, case report, colitis, programmed cell death protein-1 (PD-1)

## Abstract

**Rationale:**

As a programmed cell death 1 (PD-1) inhibitor, camrelizumab is used in the treatment of a variety of malignancies. However, a variety of immune-mediated adverse reactions have been reported in a wide range of clinical applications, including immune-related colitis, arthritis, hepatitis, etc.

**Patient concerns:**

This 56-year-old male patient experienced diarrhea, bloody stool, and knee pain after receiving camrelizumab for metastatic esophageal squamous cell carcinoma. Colonoscopy showed granular changes in the whole colonic mucosa and blurred or even disappeared vascular texture. Pathology showed chronic inflammation of the colonic mucosa. Magnetic resonance imaging of knee joint showed exudative inflammatory changes in bilateral knee joints.

**Diagnosis:**

Immune checkpoint inhibitor-induced colitis and arthritis.

**Interventions:**

Mesalazine oral (extended-release granules, 1000 mg/quarter in die daily). Dexamethasone sodium phosphate (once daily, 5mg in the evening) and compound cypress liquid (once daily, 100ml in the evening) were given by enema. Anti-inflammatory and analgesic treatment of bone pain plaster.

**Outcomes:**

The patient had diarrhea reduced to 3 times/day, no more bloody stools, and the knee pain was relieved.

**Lessons:**

This article describes the cases of immune-related colitis and arthritis caused by camrelizumab, and recommends considering the risk of colitis and arthritis with camrelizumab monotherapy or combination therapy.

## 1. Introduction

The emergence of immune checkpoint inhibitors marks a new era in cancer therapy, and their continuous evolution has brought major breakthroughs in cancer treatment.^[1]^ As the preferred treatment for advanced cancer, immune checkpoint inhibitors have achieved remarkable success in melanoma, non-small cell lung cancer, esophageal squamous cell carcinoma and other tumors.^[2]^ Programmed cell death protein-1 (PD-1) is a cell surface receptor present on T cells. As an immune checkpoint, PD-1 inhibits the transcription of related genes by specifically binding to its ligand PD-L1, thereby preventing the proliferation of T cells and ultimately leading to the loss of immune function.^[3]^ As a result, some tumors can express PD-L1 at high levels, with the aim of weakening the T cell immune response to them. Camrelizumab (AiRuiKa) is a humanized high-affinity IgG 4-κ anti-PD-1 monoclonal antibody that acts by binding to and blocking the binding of PD-1 to its ligand, PD-L1.^[4]^ However, this may lead to excessive activation of T cells, triggering T cell imbalance, which in turn leads to adverse immune responses, including immune-related reactive cutaneous capillary endothelial cell proliferation (RCCEP), colitis, arthritis, hepatitis, etc.

## 2. Case presentations

On 29-Dec-2021, a 56-year-old male was admitted to our hospital for severe diarrhea. The patient was previously diagnosed with recurrent and advanced esophageal squamous cell carcinoma and had been treated with paclitaxel (400mg/day, IV) and camrelizumab (200mg/day, IV) at a cancer specialist hospital 9 weeks earlier. After about 8 weeks, the patient began to feel diarrhea, accompanied by thin yellow stools, 20–30 times a day. The patient also experienced bilateral knee pain. He tried some antidiarrheal medication and his symptoms improved slightly, but they quickly returned after the medication was stopped. The patient was later taken to a local hospital where after treatment with levofloxacin and piperacillin sodium, the symptoms improved and the number of bowel movements decreased to 10 times per day. However, the day before admission, the patient started to have bloody stools.

After admission, we performed a series of auxiliary examinations. Laboratory tests showed elevated C-reactive protein at 19 mg/L (normal range < 5 mg/L)and decreased albumin level at 32.2 g/L (normal range 40~55 g/L). Stool analysis revealed the presence of a large number of white and red blood cells (see Tables 1 and 2 for detailed data). In addition, abdominal computed tomography (CT) revealed thickening of the large bowel wall with inflammatory exudation. We used montmorillonite powder and isopropyl ammonium bromide to reduce the number of diarrhea, glutamine granules to improve intestinal function, and probiotics to improve intestinal flora. With the progress of the above drug treatment, the patient symptoms improved significantly, the number of defecations decreased, bloody stools stopped, and abdominal pain relieved.

**Table 1:** The results of blood of the patient.

Results of blood	12/30/2021	01/03/2022	01/10/2022	01/15/2022
CRP (mg/L)	19	89.1	50.7	50.7
Hb (g/L)	132	114	120	112
PCT (ng/L)	0.061	0.072	0.315	0.147
NEUT%	0.705	0.633	0.690	0.809
WBC (×10^9)	6.54	3.67	4.53	3.51
ALB (g/L)	32.20	26.00	33.00	32.80
K + (mmol/L)	3.73	3.29	4.38	4.25

CRP, C-reactive protein (normal range < 5 mg/L); ALB, albumin (normal range 40~55 g/L); Hb, hemoglobin (normal range 130~175 g/L); PCT, procalcitonin (normal range 0~0.05 ng/mL); WBC, white blood cells count (normal range 3.5~9.5 × 10^9); NEUT%, neutrophil percentage (normal 0.40~0.75); K+, potassium ions (normal range 3.5~5.3 mmol/L); RBC, red blood cells count (normal 4.3~5.8 × 10^9); FOBT, stool occult blood test; NA, normal range.

**Table 2:** The results of fecal tests of the patient.

Fecal examination	01/01/2022	01/03/2022	01/07/2022	01/11/2022	01/13/2022
Pus cell	NA	2+/HP	1+/HP	NA	NA
RBC	2+/HP	3+/HP	2+/HP	<1+/HP	NA
WBC	4+/HP	3+/HP	3+/HP	2+/HP	NA
FOBT	Positive	Positive	Positive	Positive	Positive

CRP, C-reactive protein (normal range < 5 mg/L); ALB, albumin (normal range 40~55 g/L); Hb, hemoglobin (normal range 130~175 g/L); PCT, procalcitonin (normal range 0~0.05 ng/mL); WBC, white blood cells count (normal range 3.5~9.5 × 10^9); NEUT%, neutrophil percentage (normal 0.40~0.75); K+, potassium ions (normal range 3.5~5.3 mmol/L); RBC, red blood cells count (normal 4.3~5.8 × 10^9); FOBT, stool occult blood test; NA, normal range.

**Figure 1.**
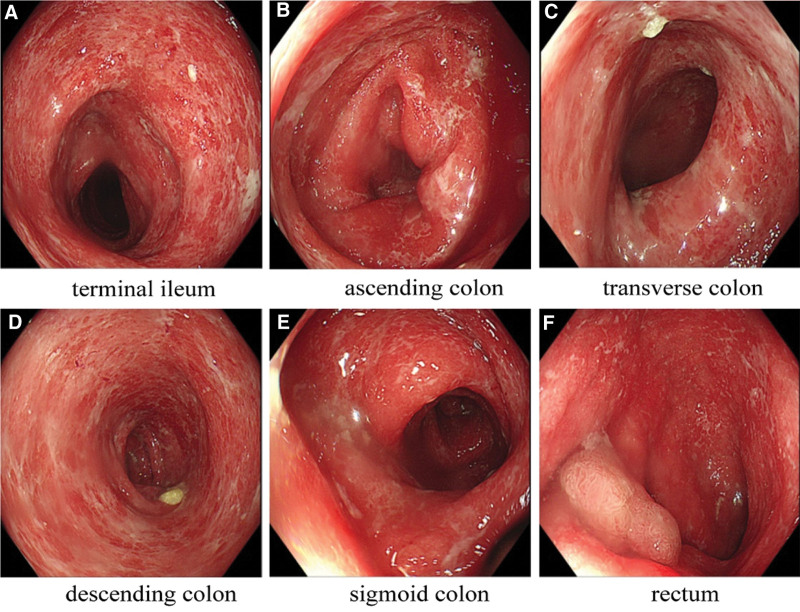
(A–F) Under colonoscopy, the entire colonic mucosa exhibited granular changes, with blurred or absent vascular texture, widely scattered patchy white plaques, and mucosal surface bleeding in some areas.

**Figure 2.**
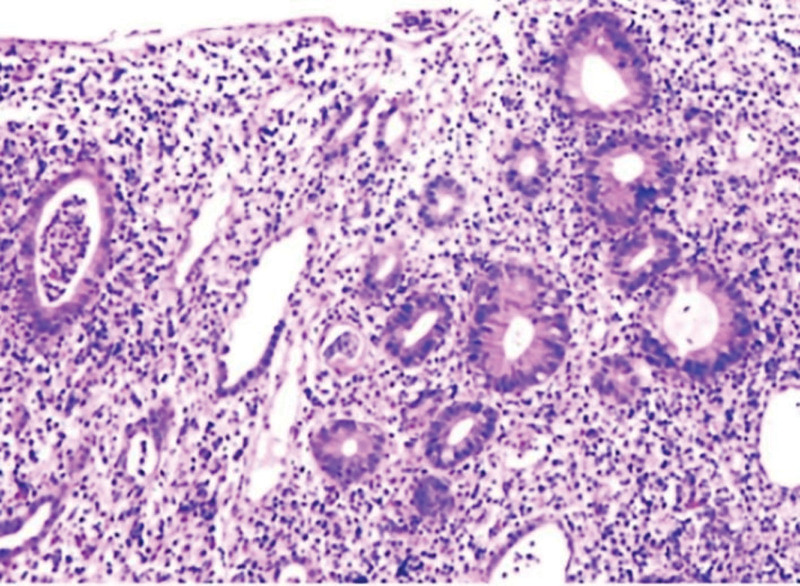
The histological pathology of the colonoscopy showed extensive mucosal erosion with severely acute and chronic inflammation which was accompanied by a small amount of eosinophil infiltration, local granulation tissue hyperplasia and a small amount of topical inflammatory exudate. In addition, crypt abscesses may be found in rectal pathology. Immunohistochemistry: Cytomegalovirus (CMV) (−), small RNA encoded by Epstein-Barr virus (EBER) (−).

However, on the 5th day of hospitalization, the patient again had bloody stools with a corresponding increase in the number of stools, and joint pain was more pronounced with an increase in C-reactive protein to 89.1 mg/L. On 05-Jan-2022, colonoscopy results showed persistent inflammation of the entire colon, which was suspected to be ulcerative colitis (see Fig. 1A–F). Colonoscopic histopathology revealed extensive mucosal erosion with severe acute and chronic inflammation, a few eosinophilic infiltrates, localized granulation tissue hyperplasia, and minimal inflammatory exudation. In rectal pathology, crypt abscesses were seen (see Fig. 2). Magnetic resonance imaging of the knee showed inflammatory exudative changes in both knees. In addition, we found no pathogenic bacteria in the patient serum and stool cultures. Cytomegalovirus and Clostridium difficile tests were negative. Based on the patient symptoms and ancillary examination results, we suspected that the patient may have developed colitis and arthritis associated with immunotherapy. Therefore, we stopped camrelizumab and started oral mesalazine (extended-release granules, 1000 mg/quater in die daily). In addition, we treated the patient with dexamethasone sodium phosphate enema (5mg once daily in the evening) and compound cypress solution enema(100ml once daily in the evening). For patients with knee pain, we used the plaster anti-inflammatory analgesic treatment. After 10 days of the above treatment, the patient clinical symptoms were significantly relieved, the number of diarrhea was reduced to 3 times/day, bloody stools stopped, and knee pain was relieved. On 17-Jan-2022, the patient was discharged. In terms of diet, we recommend that patients follow a Mediterranean diet. After discharge, the patient continued to take mesalazine sustained-release granules, steroids and analgesics.

Unfortunately, due to economic factors, the patient was not able to undergo another colonoscopy. At subsequent follow-up, the patient reported significant improvement in diarrhea symptoms, no more bloody stools, and significantly less joint pain than before.

## 3. Discussion

Studies have shown that among the immunotherapy-related adverse reactions caused by the application of immune checkpoint inhibitors, the incidence of diarrhea/colitis is 9.47%, and the incidence of Grade 3 and above diarrhea/colitis is 0.59%.^[5]^The clinical manifestation is usually diarrhea, and some patients will have bloody stools. In severe cases, it may be life-threatening due to perforation and bleeding. The incidence of rheumatic and musculoskeletal arthritis is quite different, ranging from 4% to 22%.^[6]^ The main clinical manifestations are joint pain, swelling, morning stiffness, etc. Therefore, in the case of diarrhea and bloody stool after receiving immune checkpoint inhibitor treatment, we first rule out whether the patient has a history of facial disease in the past, determine the etiology through auxiliary examination, and adopt different treatment strategies. In this case, we excluded infectious diarrhea and colitis by stool and blood tests after excluding the patient previous history of arthritis and inflammatory bowel disease. Empiric therapy was used, including mesalazine and hormone therapy. After treatment, the patient symptoms were significantly relieved.

Tumor cells can evade attack by the immune system through abnormal immune surveillance mediated by immune checkpoints, thereby gaining unlimited growth capacity. PD-1 and its ligand PD-L1 have been considered as important targets for anticancer drugs.^[7]^ Although these drugs have achieved encouraging results in cancer treatment, we must also be concerned about their possible toxic side effects. Excessive immune activation may lead to T-cell imbalance, leading to autoimmune diseases. In the study, Nancey et al found that regulatory T cells decreased and effector T cells increased in blood samples of patients with immune checkpoint inhibitor-induced colitis by flow cytometry analysis.^[8]^ A similar T cell imbalance has also been observed in patients with ulcerative colitis. In addition, studies have shown that PD-1-deficient mice may develop autoimmune diseases, which are associated with excessive immune activation.^[9]^

When classifying diarrhea and colitis, we can adopt different treatment strategies according to the severity of symptoms. For Grade 1 symptoms, immune checkpoint inhibitors can be continued and symptoms controlled. For secondary symptoms, the relevant toxicity management guidelines recommend oral corticosteroids, usually equivalent to 0.5 to 1mg/kg methylprednisolone equivalent per day, for more than 3 days. If symptoms improve, the dose of corticosteroids can be gradually reduced. If symptoms do not improve or worsen after 3 to 5 days of systemic hormone therapy, patients should receive tertiary treatment. For Grade III symptoms, patients required treatment with intravenous high-dose systemic corticosteroids. If the patient does not improve within 3 to 5 days after appropriate doses of hormone therapy, infliximab may be considered in combination.^[10]^ In addition, the NCCN (National Comprehensive Cancer Network) guidelines also mention that vedolizumab may be considered if infliximab is ineffective or contraindicated.^[11,12]^

## 4. Conclusion

In this case report, we present a case of a patient who developed colitis and arthritis after treatment with the PD-1 inhibitor camrelizumab. During treatment with PD-1 inhibitors, we need to closely monitor and be alert to the occurrence of immune-related side effects. At the same time, we need to delve deeper into the mechanisms of these drugs and work to predict risk factors for patients. Diagnostic and therapeutic strategies for immune-related colitis and arthritis need to be improved.

## Authors contribution

**Data curation:** Xin-Yu Ci.

**Supervision:** Mei-Juan Zhang.

**Writing – original draft:** Rong-Xin Xie.

**Writing – review & editing:** Yu-Bao Xue.

## References

[1] Zhou J Mao Q Li Y. Oral reactive capillary hemangiomas induced by SHR-1210 in the treatment of non-small cell lung cancer: a case report and literature review. BMC Oral Health. 2021;21:559. 34727912 10.1186/s12903-021-01901-9 PMC8561900

[2] Tang B Chi Z Chen Y. Safety, efficacy, and biomarker analysis of toripalimab in previously treated advanced melanoma: results of the POLARIS-01 multicenter phase II trial. Clin Cancer Res. 2020;26:4250–9. 32321714 10.1158/1078-0432.CCR-19-3922

[3] Westdorp H Sweep MWD Gorris MAJ. Mechanisms of immune checkpoint inhibitor-mediated colitis. Front Immunol. 2021;12:768957. 34777387 10.3389/fimmu.2021.768957 PMC8586074

[4] Markham A Keam SJ. Camrelizumab: first global approval. Drugs. 2019;79:1355–61. 31313098 10.1007/s40265-019-01167-0

[5] Martins F Sofiya L Sykiotis GP. Adverse effects of immune-checkpoint inhibitors: epidemiology, management and surveillance. Nat Rev Clin Oncol. 2019;16:563–80. 31092901 10.1038/s41571-019-0218-0

[6] Naidoo J Cappelli LC Forde PM. Inflammatory arthritis: a newly recognized adverse event of immune checkpoint blockade. Oncologist. 2017;22:627–30. 28576858 10.1634/theoncologist.2016-0390 PMC5469592

[7] Zheng Y Li L Pang Z. Progress in immune checkpoint inhibitors and their associated colitis. Gastroenterology. 2020;25.

[8] Nancey S Boschetti G Cotte E. Blockade of cytotoxic T-lymphocyte antigen-4 by ipilimumab is associated with a profound long-lasting depletion of Foxp3+ regulatory T cells: a mechanistic explanation for ipilimumab-induced severe enterocolitis? Inflamm Bowel Dis. 2012;18:E1598–600. 22069060 10.1002/ibd.21927

[9] Yamauchi R Araki T Mitsuyama K. The characteristics of nivolumab-induced colitis: an evaluation of three cases and a literature review. BMC Gastroenterol. 2018;18:135. 30170560 10.1186/s12876-018-0864-1 PMC6119262

[10] Weber JS Kähler KC Hauschild A. Management of immune-related adverse events and kinetics of response with ipilimumab. J Clin Oncol. 2012;30:2691–7. 22614989 10.1200/JCO.2012.41.6750

[11] Prieux-Klotz C Dior M Damotte D. Immune checkpoint inhibitor-induced colitis: diagnosis and management. Targeted Oncol. 2017;12:301–8. 10.1007/s11523-017-0495-4 28540478

[12] Kaneoka A Okada E Sugino H. Vedolizumab attenuates immune-checkpoint-therapy-induced infliximab-refractory colitis. Diagnostics (Basel, Switzerland). 2022;12:480. 35204571 10.3390/diagnostics12020480 PMC8870896

